# Supplementary material for: The Effects of Local Treatment of PTH(1-34) and Whitlockite and Hydroxyapatite Graft to the Calvarial Defect in a Rat Osteoporosis Model
Source: Biomedicines. 2024 Apr 8;12(4):820. doi: 10.3390/biomedicines12040820 (PMC11047906; doi:10.3390/biomedicines12040820)
Supplement: Supplementary file 1 [file biomedicines-12-00820-s001.zip › biomedicines-2917795-supplementary.pdf]

Supplementary Materials

Bone graft imaging

The images were measured using a stereoscopic microscope (SMZ745T, Nikon, Japan) to obtain the morphology of the fabricated bone graft (Figure S1(a,b)), and a scanning electron microscope (S-4700, Hitachi, Japan) after sputter coating with platinum to obtain the microstructure of the bone graft (Figure S1(c,d)).

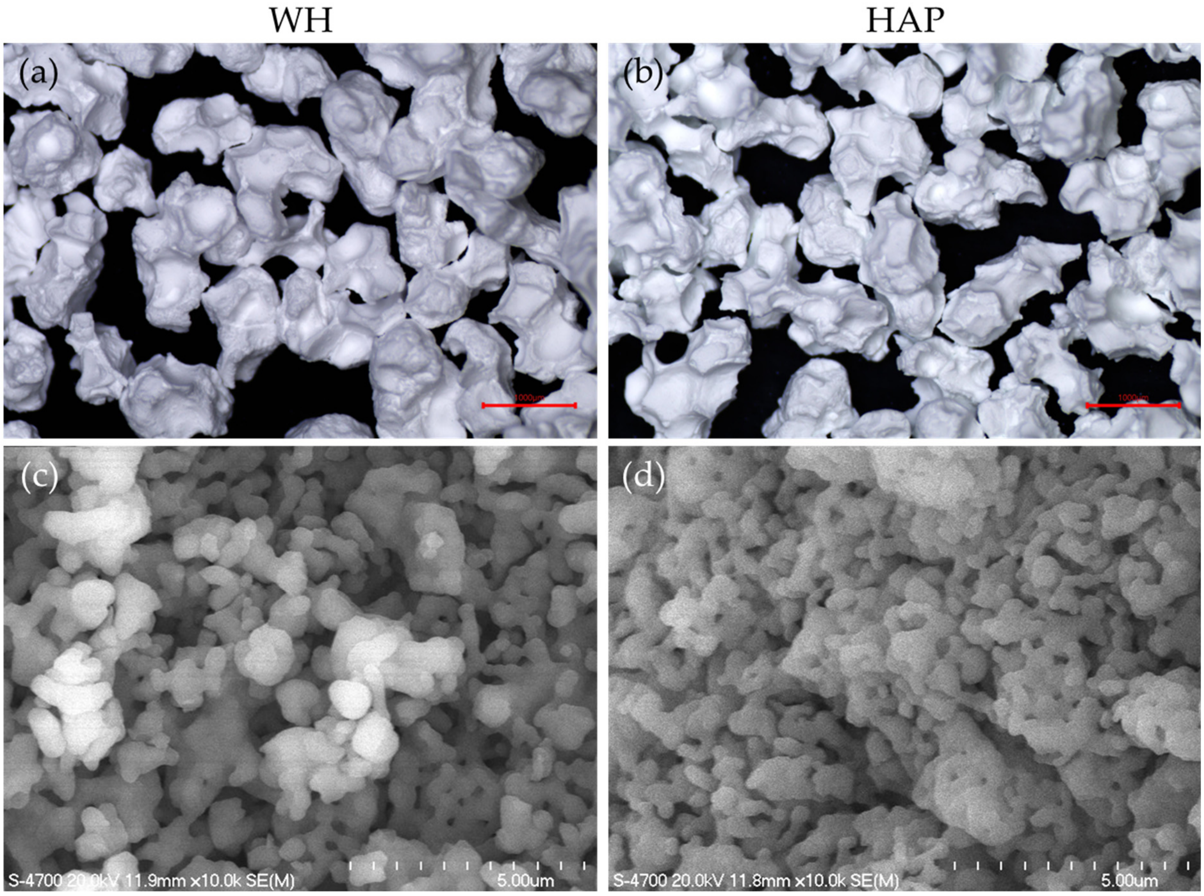

Figure S1. Stereoscopic microscope images of bone graft (a) WH bone graft, (b) HAP bone graft, (Scale bar : 1000μm). And, scanning electron microscope images of bone graft (c) WH bone graft, (d) HAP bone graft, (Scale bar : 5μm).

Wettability of bone graft

To measure the wettability of the fabricated bone graft, 1 g of bone graft was immersed in 5 ml of DW for 30 seconds and the weight change of the bone graft was measured. The measured weight was then compared to the initial bone graft weight and the amount of water the graft could hold was analyzed as $\frac{W_{final}-W_{initial}}{W_{initial}} \times 100$ (%). The analysis showed that both WH and HAP grafts were able to hold more than 100% of the graft weight in water, making them suitable for absorbing and delivering drugs to the bone defect (Figure S2).

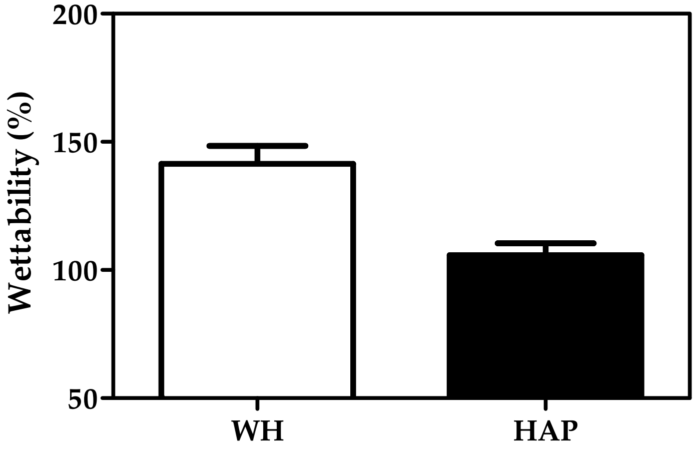

Figure S2. Wettability of WH and HAP bone graft.
